# Microbiological findings in early and late implant loss: an observational clinical case-controlled study

**DOI:** 10.1186/s12903-021-01439-w

**Published:** 2021-03-11

**Authors:** Michael Korsch, Silke-Mareike Marten, Dominic Stoll, Christopher Prechtl, Andreas Dötsch

**Affiliations:** 1 Dental Academy for Continuing Professional Development, Karlsruhe, Lorenzstrasse 7, 76135 Karlsruhe, Germany; 2 Center for Implantology and Oral Surgery, Berliner Straße 41, 69120 Heidelberg, Germany; 3 Clinic of Operative Dentistry, Periodontology and Preventive Dentistry, University Hospital, Saarland University, Building 73, 66421 Homburg, Germany; 4 Institute of Functional Interfaces, Karlsruhe Institute of Technology (KIT), Karlsruhe, Germany; 5 Department of Safety and Quality of Fruit and Vegetables, Max Rubner-Institut, Karlsruhe, Germany; 6 Department of Physiology and Biochemistry of Nutrition, Max Rubner-Institut, Karlsruhe, Germany

**Keywords:** Dental implant, Peri-implantitis, Early implant loss, Late implant loss loss, Microbial biofilm, 16 s rRNA, Bacteria

## Abstract

**Background:**

Implants are a predictable and well-established treatment method in dentistry. Nevertheless, looking at possible failures of dental implants, early and late loss have to be distinguished. The intent of the study was to report microbiological findings on the surface of implants with severe peri-implantitis, which had to be explanted.

**Methods:**

53 specimens of implants from 48 patients without severe general illnesses have been examined. The groups investigated were implants that had to be removed in the period of osseointegration (early loss, 13 patients with 14 implants) or after the healing period (late loss, 14 patients with 17 implants). The implant losses were compared with two control groups (implants with no bone loss directly after completed osseointegration, two to four months after implant placement (17 patients with 17 implants) and implants with no bone loss and prosthetic restoration for more than three years (5 patients with 5 implants)). Data about the bacteria located in the peri-implant sulcus was collected using amplification and high throughput sequencing of the 16S rRNA gene.

**Results:**

The biofilm composition differed substantially between individuals. Both in early and late implant loss, *Fusobacterium nucleatum* and *Porphyromonas gingivalis were found to be abundant*. Late lost implants showed higher bacterial diversity and in addition higher abundances of *Treponema, Fretibacterium, Pseudoramibacter and Desulfobulbus*, while microbial communities of early loss implants were very heterogeneous and showed no significantly more abundant bacterial taxa.

**Conclusions:**

Specific peri-implant pathogens were found around implants that were lost after a primarily uneventful osseointegration. *P. gingivalis* and *F. nucleatum* frequently colonized the implant in early and late losses and could therefore be characteristic for implant loss in general. In general, early lost implants showed also lower microbial diversity than late losses. However, the microbial results were not indicative of the causes of early and late losses.

## Background

The insertion of dental implants has become a routine and well predictable surgical procedure in the last decades, with high rates of osseointegration and long-term success [1]. Since the success of early implantology, this field has nowadays established itself in the daily treatment.

The reasons for tooth loss are multi-causal. Frequent problems are the development of caries at the margins of crowns, pulpal infections, periodontitis and mechanical complications, such as fractures of teeth [2–5]. While the first two factors do not affect dental implants and the rates of mechanical complications of the implant itself are low, peri-implant infections are a major risk factor for implant failure [6]. Looking at the point in time when implants are lost, early and late explantation can be distinguished.

Early implant loss takes place before prosthetic loading [7]. In those cases, osseointegration is not successful, the implant is surrounded by connective tissue and can therefore not be used to anchor the planned denture. Some authors state, that the reasons for this event may be bacterial infections, surgical mistakes like inadequate cooling during implant bed preparation or an overload of the implant by a provisional denture [7]. The only treatment option is to remove the implant and to repeat the surgical procedure, which is quite frustrating for both the patient and the surgeon. On the other hand, primary osseointegrated implants can lose their connection to the surrounding bone [6]. As a reason, bacterial infections of the peri-implant tissues have been reported in several investigations, similar to the pathogenesis of periodontitis [8]. The infection leads to an inflammatory response, causing peri-implant mucositis. If this soft tissue reaction is not controlled, the infection will lead to an inflammatory process of the peri-implant bone, resulting in bone resorption. This plaque-associated bone loss is defined as peri-implantitis [9]. Several health conditions, such as periodontitis or diabetes and smoking, have been discussed as risk factors for peri-implantitis [8, 10, 11].

In the past decades, the knowledge of bacterial infections, especially biofilms, has changed fundamentally [12, 13]. While the thesis that pathogens in periodontitis and peri-implantitis are similar had been suggested in several previous studies based on genetic analysis methods [14–16] it is meanwhile well established that there are certain differences regarding the microbiota in those diseases using new and more accurate diagnostic methods [17–22]. Also the individual reaction of the immune system and changes of the bacterial constellation around titanium implants have been discussed [23, 24]. On the other hand, some authors even state, that qualitative findings in pockets of infected implants are similar to those in healthy sites, questioning if the number of bacteria is the main reason leading to the disease [25]. New findings could either support or question the knowledge of bacterial findings around dental implants. Furthermore, there is no data regarding bacterial profiles of early lost implants, emphasizing the need of this study.

The aim of this study was to analyze the bacterial population in the peri-implant pocket of implants with severe peri-implantitis and to compare the bacterial biofilm of implants of early and late loss.

## Methods

The present clinical study investigated 53 dental implants from 48 patients. Main focus of the study was a microbial comparison of findings on the surface of implants which got lost during the time of osseointegration (early loss) and implant loss because of peri-implantitis (late loss). All results were collated by the same dentist at the Dental Academy for Continuing Professional Development (Karlsruhe, Germany).

General inclusion criteria were: implants with bleeding on probing (BOP) in combination with severe loss of the bone-to-implant contact, either in the period of healing after the insertion (< 3 months, “early loss”) or after uneventful osseointegration and loading (> 3 years, “late loss”). Exclusion criteria were: patients aged younger than 18 years, radiation or bisphosphonate therapy, patients with untreated periodontitis and severe general conditions, such as uncontrolled diabetes (HbA_1c_ > 58 mmol/mol / > 7,5%), tumors, severe heart disease or a reduced state of general health. Patients who had taken antibiotics within the last two months prior to sample collection were excluded. Smokers were not excluded from the study. Included were only patients with one or more implants which had been inserted in our clinic. Only implants with severe peri-implant bone loss and no chance of regeneration or preservation were included. Implants that cannot be preserved, due to severe peri-implantitis, were defined as having increased peri-implant pocket depth in combination with bleeding on probing, persistent symptoms and radiological bone loss of at least 6 mm. Also specimens from implants without peri-implantitis (no bone loss), BOP and pocket suppuration were integrated into the study as a control group.

Implants with peri-implantitis and bone loss, but chances of preservation were excluded.

Study population.

Patients were divided into four groups:

27 patients with severe peri-implantitis and implants without a chance of preservation were divided in two groups.

- Group E: early implant loss (implants with severe peri-implantitis during osseointegration prior to prosthetic restoration, ≤ 3 months after implant placement), 13 patients with 14 implants
- Group L: late implant loss (implants with severe peri-implantitis and prosthetic restoration for more than three years), 14 patients with 17 implants (three patients with two implants)
  For a comparison to the healthy oral situation we created two control groups:
- Group CE: control group (implants with no bone loss, no BOP and no pocket suppuration directly after completed osseointegration, two to four months after implant placement), 17 patients with 17 implants
- Group CL: control group (implants with no bone loss, no BOP and no pocket suppuration and prosthetic restoration for more than three years), 5 patients with 5 implants

Note that one patient contributed two implants to the late loss group (L) and an additional implant to group CE, effectively being counted twice in the number of patients per group. Three additional patients were initially recruited but not included in the analysis because they not fully met the inclusion criteria.

The implants were placed by three different surgeons with several years of experience.

The observational study was approved by the ethical review committee of the local medical association (Institutional Review Board of the Saarland Medical Council, Germany; ID: 232/12) and was conducted in accordance with the Declaration of Helsinki and the Professional Code for Physicians of the local Medical Council**.** All patients were informed about the purpose of the study by the examiner and signed a form of consent.

Clinical procedure of documentation of clinical findings and sampling.

**Fig. 1:**
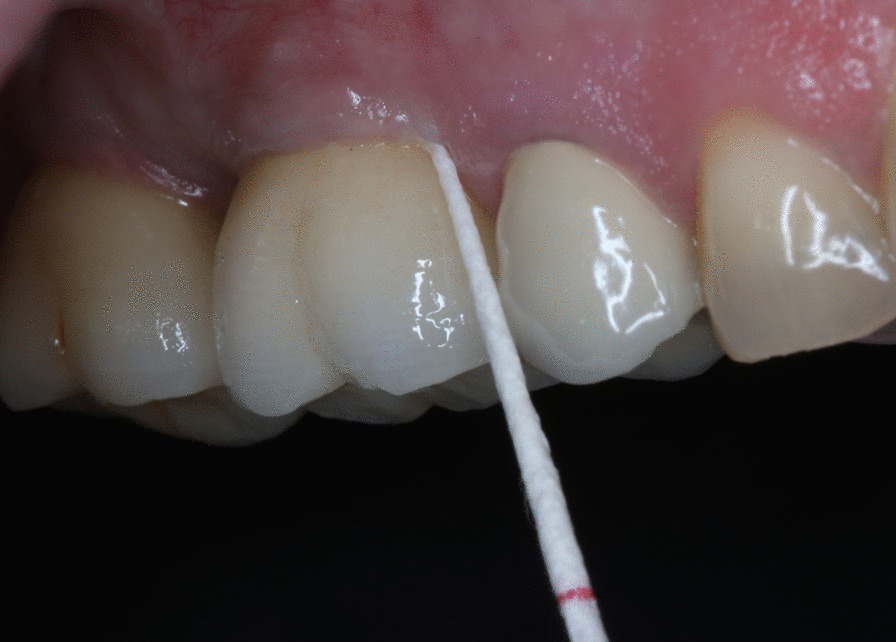
The figure shows a sample which was taken with sterile paper points from the peri-implant sulcus around infected or healthy implants

- Patients were asked about their case history, nicotine abuse, diabetes, regular use of mouth rinses (at least once per week) and antibiotics in the past 12 months, and if periodontitis had been diagnosed.
- In each patient a pool sample was taken with three sterile paper points from the peri-implant sulcus around infected or healthy implants. (Fig. 1). The supramucosal plaque was removed with a cotton swab prior to submucosal sample collection. The surface of the denture was then air-dryed using the multifunctional syringe. Only after these measures were taken was the submucosal sample taken with a paper point. These measures prevented supra- and submucosal plaque from being mixed during sample collection.
- Any existing signs of inflammation (pocket suppuration) were documented. In patients with multiple implants, samples were collected for each implant separately, but the data were later pooled to prevent an unbalanced dataset.
- If a severe peri-implantitis was diagnosed, explantation was done under local anaesthesia with articaine with epinephrine 1:100.000 (Citocartin Sopira®, Heraeus Kulzer GmbH, Hanau, Germany).

All patients were informed in advance about the clinical procedure to be performed. In addition, the patients were informed about obtaining paper point samples for a future microbial analysis in connection with the study. All patients gave their informed consent in writing.

For the microbial analysis of the peri-implant samples the bacterial DNA from the paper point of a total of 53 implants from 48 patients were obtained and analyzed for taxonomic composition by sequencing the V1-V2 variable regions of the 16S rRNA gene. The sequencing was performed in the Max Rubner-Institut, Karlsruhe, Germany.

The paper points were applicated for 20 s, following the established clinical routine for sample collection of peri-implant pathogens [26].

### Evaluation at patient level

Samples were obtained from each implant with three sterile paper points and then pooled. The data acquisition and microbiological evaluation took place at the implant level. For some patients multiple implants were analysed. In these cases, the microbial data were later pooled for the statistical analysis, effectively creating an average microbial composition of the implants obtained from the same patient.

One patient had samples from two groups (L and CE). In this case, the samples from the different groups were not pooled. The evaluation was therefore carried out at patient level.

### Extraction of microbial DNA

Sterile paper points were used to collect biofilm samples of the peri-implant sulcus for microbiota analysis as described previously [26]. The implants were also collected after explantation and their microbiota was likewise analyzed for control purposes. Briefly, biofilm microbes were resuspended in nuclease free water by subjecting paper points (or implants) to a combination of shaking and sonication. The suspension was centrifuged, pellets were stored at − 80 °C and then used for DNA extraction using commercial extraction protocols for genomic DNA (QIAamp Mini Kit, Qiagen, Hilden, Germany). The collected pellets of the supernatant were treated with 180 µL lysozyme solution (20 mg/mL, SIGMA-Aldrich, Taufkirchen, Germany; 20 mM Tris–HCl, pH 8.0, 2 mM EDTA, 1.2% Triton) under shaking at 37 °C for 2:15 h, followed by proteinase K digestion (20 µL proteinase K and 200 µL buffer AL) for 1:15 h under shaking at 56 °C. Finally, the DNA was eluted with 100 µL PCR-clean water and the concentration was quantified using the NanoDrop equipment (PEQLAB, Erlangen, Germany). One empty extraction without any sample material was used a control for background DNA contaminations (contamination control).

### Illumina sequencing of amplicons targeting the 16S rRNA gene

Amplicons of the V1-V2 region of the bacterial 16S rRNA gene were prepared as published elsewhere [27]. Briefly, the genomic sequence of the 16S rRNA gene was amplified with primers that were derived from the previously described primers 27F and 338R [28, 29] and contained sequences compatible with Illumina sequencing platforms and a 6-nt barcode sequence. The resulting DNA was used as template for a second PCR, using primers designed to introduce full-length Illumina adapter sequences including Illumina 6-nt index sequences to enable high-level multiplexing. To control for potential DNA contamination an additional sample was amplified without template DNA (contamination control sample). Libraries were pooled and subjected to 250 nt paired-end sequencing on an Illumina MiSeq machine. For data analysis, the obtained raw reads were first demultiplexed based on barcode sequences and primer sequences removed using a Perl script, thereby removing reads that did not contain the primer sequence. Further, the reads were processed using *dada2* version 1.16.0 [30]. *Dada2* determines *amplified sequence variants* (ASVs) by removing sequencing errors, effectively denoising the data, which tends to produce more accurate results than the commonly used *operational taxonomic units* or OTUs [31]. Briefly, sequence reads were trimmed to a length of 200 nt (forward read) and 150 nt (reverse read) and 5 nt of the left end were trimmed additionally. Reads with ambiguous base calls and an expected error rate larger than 2 were discarded and ASVs were inferred using the *pseudo-pooling* algorithm. Chimeric sequences were removed and ASVs classified using the RDP Naïve Bayesian Classifier algorithm as implemented in *dada2* against the Silva database v138 [32]. Were possible by exact matching, ASVs were assigned species names using the *assignSpecies* function of *dada2*. After the preprocessing the data amounted to between 1305 and 205,073 sequences per sample.

### Statistical analysis of microbiome data

Further analysis of the microbiome data was done in R version 3.6.1 [33] using packages *phyloseq* [34], *vegan* [35] and *ggplot2* [36]. The contamination control sample yielded 397 reads and 13 ASVs represented by more than 5 sequences in the contamination control were removed from all samples. Alpha-diversity of the sampled biofilms was estimated using the Inverse Simpson Index, calculated by the *phyloseq* function *estimate_richness*. Principal coordinates analyses (PCoA) were performed using the *ordinate* function with Jenson-Shannon Divergence (JSD) as distance metric. Differences of single variables between groups of samples were statistically tested using the Kruskal–Wallis Rank Sum Test (R function *kruskal.test*) for multiple groups and Wilcoxon Rank Sum Test (R function *pairwise.wilcox.test*) for pairwise comparisons of groups with the Benjamini–Hochberg adjustment to control false discovery rate in multiple comparisons [37]. Differences in the microbiota composition (beta-diversity) were tested by performing Permutational Multivariate Analysis of Variance (PERMANOVA) on the JSD matrix, using the *adonis* function of the R package *vegan* [35] and *pairwiseAdonis* by Martinez Arbizu & Monteux [38]. Differential abundances of species were calculated by merging ASVs that were classified to belong to the same species (or genus for those ASVs that could not be classified at the species level) and by comparing two groups with the R package *ALDEx2* v1.20.0 [39] using the functions *aldex.ttest* and *aldex.effect*. Species were defined as differentially abundant, if the p-value of the Welch test was less than 0.05.

The study was conducted in accordance with the STROBE guidelines (https://www.strobe-statement.org).

## Results

### Composition of the peri-implant microbiota

Across all samples, 552 taxa (amplified sequence variants, ASVs) were detected that occurred with an abundance of at least 1% in one or more samples. Most of the identified taxa belonged to the six phyla *Actinobacteriota, Bacteroidota, Firmicutes, Fusobacteriota, Proteobacteria* and *Spirochaetota*. All samples could be analyzed and were included in the study.

**Fig. 2:**
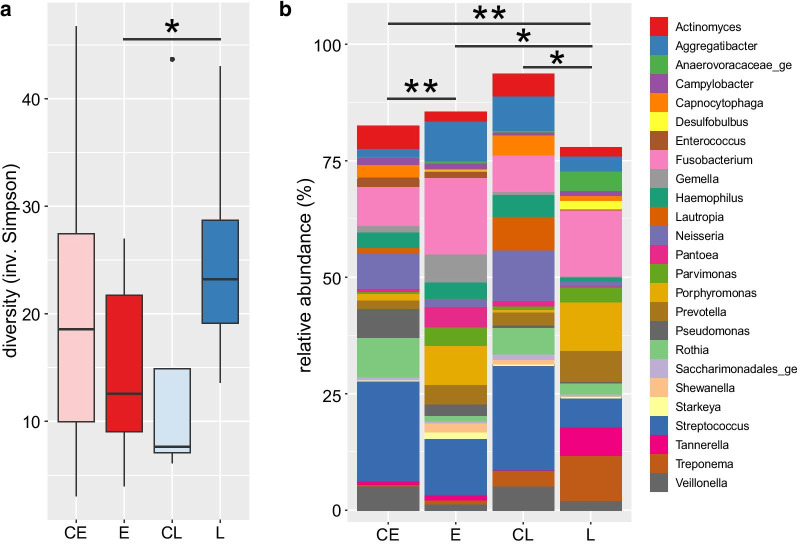
**a** Alpha-diversity of microbial composition in samples of implant groups. The groups of implants are labelled CE—early control, E—early implant loss, CL—late control, L—late implant loss. Diversity was calculated using the inverse Simpson index and is here depicted as box-and-whiskers-plots with the box representing values between the 1st and 3rd quartile, a black line indicating the median. b) Average relative abundance of the most abundant genera across implant groups. All sequence variants (ASVs) that were classified to belong to the same genus were aggregated. Horizontal lines indicate a significant difference in the microbiota composition between groups (pairwise Wilcoxon and pairwise PERMANOVA analysis in **a**, **b**, respectively). Asterisks in both panels indicate significance levels: *p < 0.05, **p < 0.01)

**Fig. 3:**
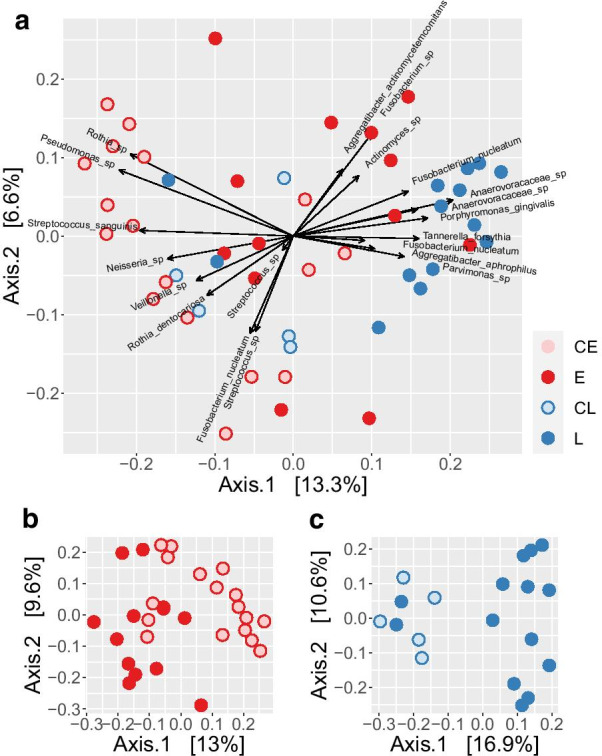
Principal coordinates analysis (PCoA) of relative abundances of ASVs across all samples. Individual samples are depicted as circles, colored by group of implants as labelled: CE—early control, E—early implant loss, CL—late control, L—late implant loss. **a** Biplot showing also arrows that represent the “loadings”, i.e. the contribution of the 20 most abundant sequence variants (ASVs) and are labelled with the species classification (if available). Axis.1 and Axis.2 represent the first two principal coordinates with the fraction of total variance explained by each coordinate written in square brackets. **b**, **c** PCoA plots of the early (E) and late (L) loss group of implants with their respective control groups

**Fig. 4:**
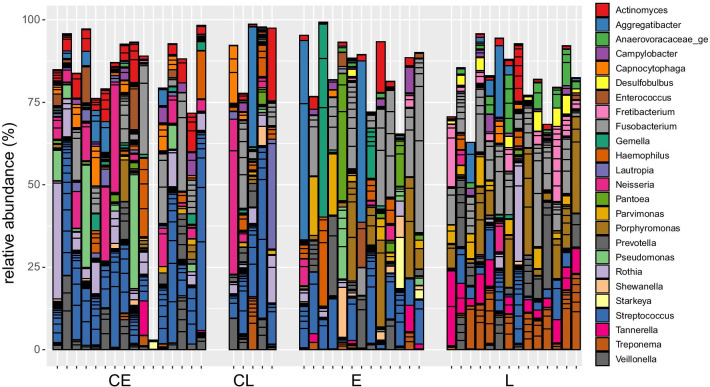
Relative abundance of the 25 genera with the highest abundance across all samples. Each column represents the composition of one patient. Columns were grouped according to the implant group: CE—early control, E—early implant loss, CL—late control, L—late implant loss. Within a group the columns were sorted according to their location on the 1st axis of the PCoA plot (Fig. 3a)

The microbial composition of the peri-implant biofilm samples showed substantial variation between individual patients, most ASVs were not found in all patients, even when comparing only patients of the same group (Fig. 2). Representatives of bacterial genera that were found in most samples (though at variable abundance) included *Streptococcus* and *Fusobacterium*. Most of the *Streptococcus* ASVs could not be classified at the species level with a few exceptions like *S. sanguinis* and *S. canis*. Some taxa were found rarely or not at all in most samples but were found at high abundances in single samples. This included extreme cases, were ASVs of a single genus (*Rothia*, *Aggregatibacter* and *Gemella)* comprised more than 50% of the sequences. Despite the large variability, a comparison of the composition across all samples revealed a pattern that partially matched the different groups of patients (Figs. 2, 3). Most notably, most of the patients who lost their implants (groups E and L) could be distinguished from their respective control groups (Fig. 3b, c). While the samples of group L clustered together on the right side of the plot (with two exceptions), the samples of group E showed larger differences. Some of the latter clustered with the L samples, some with the control samples. The difference in the composition of the different groups was found to be statistically significant, either when all groups were compared with each other or only the implant loss groups with their respective controls (p < 10^–5^, PERMANOVA Test on the beta-diversity). Patients with implant loss showed increased average abundances of *Fusobacterium* and *Porphyromonas* (Fig. 2b) and the most abundant species of these genera, *F. nucleatum* and *P. gingivalis* contributed strongly to the placement of the respective samples in the PCoA plot (Fig. 3). The two healthy control groups on the other hand harbored comparatively high abundances in bacteria of the genera *Streptococcus*, *Neisseria*, *Rothia* and *Veillonella* (Figs. 2b, 4). So far, the analysis compared the microbial compositions based on beta-diversity, thus on a global level. We also compared the data on the level of species to give further insights into the differential abundance of certain bacteria under different conditions. Comparison of the late loss groups with each other and their respective control groups results in a limited list of differentially abundant species (Table 1). Five species were found to be significantly higher in group L than in E, namely *Desulfobulbus* sp.*, Pseudoramibacter alactolyticus*, *Treponema* sp., *Fretibacterium* sp. and a species of *Anaerovoracaceae,* but the effects were quite small. Comparing groups E and L with their controls, there were 3 and 10 species found to be differentially abundant, respectively. Some bacteria were found to be differentially abundant in two of these comparisons, *Desulfobulbus* sp*.*, *P. alactolyticus* and *Fretibacterium*, all of which were found to be higher in late loss group L compared to the control group CL. In addition, *Streptococcus sanguinis* was found to be reduced in E compared to the control CE, while *Streptococcus* sp. was reduced in L compared to CL.

**Table 1:** Differential abundance of Species between different groups

Species name	FDR	Abund L	Abund E	Effect
Pseudomonas sp	0.0070	1.86	6.50	− 1.18
Anaerovoracaceae uncl	0.0240	8.82	4.07	0.94
Desulfobulbus sp	0.0142	8.82	− 0.44	1.15
Pseudoramibacter alactolyticus	0.0079	7.23	− 0.37	1.21
Treponema sp	0.0151	10.48	1.62	1.04
Pantoea sp	0.0064	2.69	7.89	− 1.13
Fretibacterium sp	0.0170	9.21	2.00	1.02
Shewanella sp	0.0078	2.75	7.55	− 1.09

Species name	FDR	Abund L	Abund CL	Effect
Rothia sp	0.0187	2.41	10.56	− 1.06
Streptococcus sp	0.0063	9.20	12.48	− 1.52
Desulfobulbus sp	0.0209	8.79	− 1.74	1.73
Pseudoramibacter alactolyticus	0.0256	7.18	− 1.40	2.10
Fretibacterium sp	0.0555	9.18	− 0.55	1.55
Haemophilus parainfluenzae	0.0015	2.36	8.62	− 1.65
Haemophilus sp	0.0035	2.63	9.32	− 1.52
Dialister pneumosintes	0.0333	4.93	− 1.98	1.43
Capnocytophaga sp	0.0930	2.14	7.00	− 0.94
Granulicatella sp	0.0299	− 0.09	5.87	− 1.12

Species name	FDR	Abund E	Abund CE	Effect
Streptococcus sanguinis	0.0061	3.69	9.99	− 1.40
Lautropia mirabilis	0.0539	1.60	7.90	− 1.00
Actinomyces naeslundii	0.0961	0.43	6.26	− 0.84

abund X: relative abundance of species in group X (clr transformed)

FDR: False discovera rate (Benjamini Hochberg corrected P value, Welch-test)

effect: ratio of median between-group differences and within-group differences

uncl.: indicates that this taxon was not classified on a lower rank

A number of parameters were collected for each patient, some of which could impact on the microbial composition (Additional file 1). Firstly, these parameters were tested if they were evenly distributed among the groups. The parameters gender, implant region, diabetes, antibiotic use (within 12 to 2 months prior to sampling), and regular mouthwash showed no significant deviation from an even distribution (p > 0.05, Fisher’s exact test). The type of implant system used showed some bias however, with the majority of implants being of type “Astra”, which were overrepresented in groups E and CE (p = 0.040). Also, smoking was unevenly distributed (p = 0.048) with smokers being overrepresented in groups E and L (77 and 78%, respectively) as compared to CE and CL (35 & 40%). Another important factor is periodontitis, which was diagnosed if found anywhere in a patient and not only close to the implant that was analyzed. Patients with implant loss had a higher incidence of periodontitis (E: 64%, L: 71%) than the control groups (CE: 29%, CL: 20%). The impact of these potential confounders on the microbial data was tested by adding each one as an independent variable to the PERMANOVA Test on beta-diversity between groups. Antibiotic use and periodontitis both had no significant effect on beta-diversity, while the differences between groups still were significant in the extended models. Likewise, the parameters diabetes, implant system smoking and regular mouthwash had no significant effect.

Biofilm samples were also analyzed directly from implants after the explantation and compared to the corresponding paper point samples to validate the results. The differences in the microbial composition were highly similar between implant and paper point samples. Although a multivariate analysis of the composition (PERMANOVA) did show a significant difference between implants and paper points (R^2^ = 0.021, p = 0.016), it was small compared to the difference found between the groups (R^2^ = 0.107, p < 10^–4^).

## Discussion

In the last decades several risk factors for the failure of dental implants have been reported – most of them being clinical aspects of the implant treatment, medications or health conditions. Looking at the clinical risks for bone loss around implants, surgical skills or mistakes regarding the surgical protocol, such as dull burrs, high pressure while drilling or high insertion torque, different healing protocols or excess of cement can cause problems [40–43]. On the other hand, medications, such as bisphosphonates or antidepressants can be risk factors for infections or implant loss [44–46]. Additionally, certain conditions, such as poor bone quality, periodontitis, bruxism, overload or mechanical complications and smoking habits can have an impact on osseointegration and long-term success of dental implants, while controlled diabetes, osteoporosis or the age of the patient do not seem to be risk factors [47–54].

Among those reasons for implant failure, bacterial infections have been reported to be a major risk factor for early and late loss of dental implants [7, 52, 55, 56]. Most of the studies focus on the microbial findings in peri-implantitis, while there is no data for early loss. Differences between research groups could be explained by different test systems and by the steady development of microbial tests. The microbial findings in peri-implantitis resemble that of periodontitis, with some notable differences [17, 19, 20, 56]. Although certain known periodontal pathogens may also be associated with the etiology of peri-implantitis, apparently there were many differences between these two clinical conditions, involving distinct microorganisms [20].

The results obtained in this study present a very heterogeneous picture of the microbes found around failed dental implants. A huge majority of this variation can likely be attributed to differences between patients that have no association with any clinically relevant parameters. Overall, the statistical analysis proved difficult due the huge underlying variation and the limited sample size of the study groups, resulting in only few bacterial groups showing differential abundances between groups that were statistically significant. Nevertheless, both early and late loss samples showed some similarity and on average could be distinguished from their respective control groups (Fig. 3). Thereby, the group of late loss samples (L) formed a more compact group with the exception of two samples, while early loss samples appeared to be more heterogeneous.

Our findings show that in the group of late lost implants pathogens such as *Tannerella forsythia* and *Treponema* sp., were frequently found around implants, which had to be removed because of severe bone loss after a primarily uneventful osseointegration. Additionally, *Porphyromonas gingivalis* and *Fusobacterium nucleatum* were found in early and late loss, although the latter was found in several variants all classified as the same species, only some of which contributed to the communities in the late loss samples. The statistical analysis also revealed increased abundances of *Desulfobulbus* sp., *Fretibacterium* sp. and *Pseudoramibacter alactolyticus* which were also previously found in peri-implant sites [57, 58].

For early loss of implants, the microbial results were very heterogeneous and only a subset of these cases were similar to the major part of late loss samples, while a large fraction resembled the healthy controls. Some of the samples in this group were each dominated by a single genus, notably *Fusobacterium*, *Aggregatibacter* and *Gemella* that accounted for more than 50% of the microbial sequences found in these samples (Fig. 4). Such samples might partially explain the observation that implant lost early had a lower microbial diversity (Fig. 2a) than the late loss implants. This heterogeneity between patients points towards multiple potential risk factors contributing to early implant losses, which might include i) characteristic microbial communities, ii) dysbiosis involving other oral bacteria and iii) other, non-microbial causes like surgical mistakes or poor bone quality. Studies on the early bacterial colonization of healthy implants show a biofilm similar to that of remaining teeth [59–61]. Especially *Streptococcus* as an early colonizer is commonly found in high abundance [59–61], which is also reflected in our own data. A comparison in the early healing phase of implants of patients with chronic periodontitis and volunteers without periodontitis showed that the presence of bacterial species associated with periodontitis was always higher in the chronic periodontitis group; the difference was significant for *Porphyromonas gingivalis* and *Aggregatibacter actinomycetemcomitans* [62]. It should be noted that these bacteria are commonly found in the context of healthy implants in low numbers and their mere presence is not indicative of periodontitis [61, 63, 64].

The use of antibiotics is included in many implantological protocols. This approach has an impact on the oral microbiome [65]. Therefore, patients who had taken antibiotics within the last two months prior to explantation were excluded. However, about half of the patients used antibiotics within 12 to 2 months prior to sampling. We therefore tested the effect of antibiotics on the microbial composition but found no differences between patients with and without the use of antibiotics.

In the present study, regular use of mouthwash did not show any significant impact on the peri-implant microbiome. It is known that mouthwash affects the supragingival biofilm [66–68]. However, it is unclear which kind of affect a mouthwash with common rinsing solutions has on the submucosal biofilm of implants. The heterogeneity within the groups and the small number of cases may have had an influence on the present results. There was a number of additional potential confounding variables that might have affected the microbial composition in the patients examined: use of diabetes, smoking, differences in the implant system and especially periodontitis. The distribution of smokers and implant systems was indeed biased between the different groups. Smokers were overrepresented in both groups with implant loss and less present in the control groups but the microbial composition showed no significant difference. This might hint towards an effect of smoking on the outcome, which is independent from the microbial colonization. The implant systems were biased with the System Astra being overrepresented in the “younger” implants of groups E and CE, which might be due to the fact that these implants were placed by the same surgent. Astra was the main implant system in this clinic at the time. Most other implant systems were represented only in few numbers, so we cannot draw conclusions on this factor and besides, the microbial composition was not different between the different systems. A potentially important factor for the occurrence of inflammation and source of oral pathogens is the diagnosis of periodontitis. The observation that patients with implant loss were more likely to also be diagnosed with periodontitis than those of the control groups indeed hints that it could be an important contributing factor. Notably, we nevertheless found no significant difference in the composition of the implant-associated biofilm in patients with periodontitis, although some periodontal pathogens were found to be abundant in patients with implant loss. In a mixed model with periodontitis and the experimental group as independent factors, the microbial composition was found to be different between the groups but not between patients with and without periodontitis. Thus, although periodontitis seems to be a potential factor for implant failure, the composition of the implant-associated biofilm appears to be independently associated.

Because of the sample size, the data presented should be interpreted as a first indication, especially regarding to the heterogeneous findings in early implant loss. It can also not be distinguished, if the bacterial colonization is the reason for the loss of the implant, or if it represents a secondary infection after osseointegration failed or was lost. This underlines the importance of further studies that include a higher number of implants that also assess the different potential risks of early losses.

## Conclusions

Summarizing, samples from late lost implants formed a more homogeneous group in terms of their microbial composition, while early loss samples appeared to be more heterogeneous. In general, early lost implants showed also lower microbial diversity than late losses. However, the microbial results were not indicative of the causes of early and late losses.

## Supplementary Information

**Additional file 1.** Table of patient metadata.

## Data Availability

The raw sequence data were uploaded to the European Nucleotide Archive (ENA, https://www.ebi.ac.uk/ena/browser/view/PRJEB41299) with the accession No. PRJEB41299.

## Abbreviations

ASVs: Amplified sequence variants
BOP: Bleeding on probing
DNA: Deoxyribonucleic acid
FDR: False discovera rate
Group CE: Control group (implants with no bone loss, no BOP and no pocket suppuration directly after completed osseointegration)
Group CL: Control group (implants with no bone loss, no BOP and no pocket suppuration and prosthetic restoration for more than three years)
Group E: Early implant loss
Group L: Late implant loss
JSD: Jenson-Shannon Divergence
OTUs: Operational taxonomic units
PCR: Polymerase chain reaction
PCoA: Principal coordinates analyses
PERMANOVA: Permutational Multivariate Analysis of Variance
rRNA: Ribosomal ribonucleic acid

## Availability of data and materials

We provide the datas as supplementary files. The raw sequence data were uploaded to the European Nucleotide Archive (ENA, https://www.ebi.ac.uk/ena/browser/view/PRJEB41299) with the accession No. PRJEB41299.
